# The Epigenetic Regulator CXXC Finger Protein 1 is Essential for Murine Hematopoiesis

**DOI:** 10.1371/journal.pone.0113745

**Published:** 2014-12-03

**Authors:** Kristin T. Chun, Binghui Li, Erika Dobrota, Courtney Tate, Jeong-Heon Lee, Shehnaz Khan, Laura Haneline, Harm HogenEsch, David G. Skalnik

**Affiliations:** 1 Herman B Wells Center for Pediatric Research, Department of Pediatrics, Indiana University School of Medicine, Indianapolis, Indiana, United States of America; 2 Department of Biochemistry & Molecular Biology, Indiana University School of Medicine, Indianapolis, Indiana, United States of America; 3 Department of Comparative Pathobiology, Purdue University, West Lafayette, Indiana, United States of America; 4 Biology Department, Indiana University-Purdue University Indianapolis School of Science, Indianapolis, Indiana, United States of America; 5 Departments of Microbiology & Immunology and Cellular & Integrative Physiology, Indiana University School of Medicine, Indianapolis, Indiana, United States of America; Rutgers - New Jersey Medical School, United States of America

## Abstract

CXXC finger protein 1 (Cfp1), encoded by the *Cxxc1* gene, binds to DNA sequences containing an unmethylated CpG dinucleotide and is an epigenetic regulator of both cytosine and histone methylation. *Cxxc1-null* mouse embryos fail to gastrulate, and *Cxxc1-null* embryonic stem cells are viable but cannot differentiate, suggesting that Cfp1 is required for chromatin remodeling associated with stem cell differentiation and embryogenesis. Mice homozygous for a conditional *Cxxc1* deletion allele and carrying the inducible *Mx1-Cre* transgene were generated to assess Cfp1 function in adult animals. Induction of Cre expression in adult animals led to Cfp1 depletion in hematopoietic cells, a failure of hematopoiesis with a nearly complete loss of lineage-committed progenitors and mature cells, elevated levels of apoptosis, and death within two weeks. A similar pathology resulted following transplantation of conditional *Cxxc1* bone marrow cells into wild type recipients, demonstrating this phenotype is intrinsic to Cfp1 function within bone marrow cells. Remarkably, the Lin^−^Sca-1^+^c-Kit^+^ population of cells in the bone marrow, which is enriched for hematopoietic stem cells and multi-potential progenitor cells, persists and expands in the absence of Cfp1 during this time frame. Thus, Cfp1 is necessary for hematopoietic stem and multi-potential progenitor cell function and for the developmental potential of differentiating hematopoietic cells.

## Introduction

Hematopoiesis is a dynamic process in which multipotent hematopoietic stem cells (HSCs) give rise to all lineages of mature blood cells. HSC differentiation is balanced with self-renewal, which maintains a pool of stem cells that sustain hematopoiesis for the lifespan of the organism [1], [2]. How this balance is controlled at the molecular level remains unclear, but regulation of chromatin structure clearly plays a critical role. Indeed, altered patterns of epigenetic modifications have been linked to HSC aging [3], and epigenetic reprogramming can reset aged HSCs into a young state [4]. Furthermore, stem cell lineage commitment and cellular differentiation involve global remodeling of chromatin structure and a progressive accumulation of heterochromatin and restriction of gene expression and developmental potential [5], [6].

Mammalian CXXC finger protein 1 (Cfp1), encoded by the *Cxxc1* gene, is an important epigenetic regulator that interacts with DNA sequences containing an unmethylated CpG dinucleotide [7], [8]. Cfp1-deficient embryonic stem (ES) cells are viable but fail to differentiate in vitro [9]. These ES cells exhibit a 70% loss of global genomic cytosine methylation and reduced maintenance DNA methytransferase (Dnmt) activity. Cfp1 physically interacts with Dnmt1 [10], and cells lacking Cfp1 express reduced levels of Dnmt1 protein due to reduced Dnmt1 half-life and translation efficiency [11]. Mouse embryos lacking Cfp1 exhibit a peri-implantation death and fail to gastrulate [12]. Cfp1 is also a component of the Setd1A and Setd1B histone H3-Lys4 methyltransferase complexes [13], [14]. Ablation of the murine gene encoding either Setd1A or Setd1B leads to embryonic lethality, and Setd1A is also required for ES cell viability and for the derivation of induced pluripotent stem cells [15].

*Cxxc1*-null ES cells contain elevated levels of histone H3-Lys4 methylation and reduced levels of histone H3-Lys9 methylation [13]. This is consistent with the observed reduction in genomic cytosine methylation, and suggests a reduction in the level of heterochromatin in cells lacking Cfp1. Cfp1 appears necessary to restrict the activity of the Setd1 histone methyltransferase complexes, as both Setd1A protein and histone H3-Lys4 methylation inappropriately partially co-localize with heterochromatin in the absence of Cfp1 [16]. Consistent with this finding, chromatin immunoprecipitation studies found that Cfp1 is bound to 80% of unmethylated CpG islands in vivo, and depletion of Cfp1 leads to loss of histone H3-Lys4 tri-methylation at these sites [17]. Thus, Cfp1 appears to serve as a reader of cytosine methylation patterns and to tether the Setd1 histone H3-Lys4 methyltransferases to appropriate sites in the genome. The global increase in histone H3-Lys4 methylation following depletion of Cfp1, despite its loss at CpG islands, could therefore be explained by the failure of Cfp1 to restrict Setd1 localization to CpG islands, and the inappropriate drifting of methyltransferases throughout the genome. Thus, Cfp1 is a critical regulator of both cytosine methylation and histone methylation, and may function to integrate these epigenetic modifications during development. Surprisingly, targeting of histone H3-Lys4 tri-methylation to CpG islands is largely rescued upon introduction of a mutated form of Cfp1 that lacks DNA-binding activity [18], suggesting that the mechanism of Cfp1 action is more complex than originally hypothesized.

Mouse embryos lacking Cfp1 exhibit a peri-implantation death and fail to gastrulate [12], demonstrating that Cfp1 is essential for embryonic development. Furthermore, injection of zebrafish embryos with antisense morpholino oligonucleotides specific to the Cxxc1 transcript leads to embryo runting, cardiac edema, and a failure of primitive hematopoiesis [19]. These gross zebrafish developmental abnormalities are associated with a 60% decline in global genomic cytosine methylation levels [19]. In addition, shRNA-mediated depletion of Cfp1 inhibits the survival and differentiation capacity of human myeloid cell lines [20]. Thus, Cfp1 is not only required for early developmental processes but is also required for post-gastrulation vertebrate development. Given that Cfp1 is a regulator of both cytosine methylation and histone methylation, it is intriguing that the developmental timing of *Cxxc1-null* mouse embryo death coincides with a time of global epigenetic remodeling, raising the possibility that Cfp1 plays a role in regulating this wave of epigenetic reprogramming.

The early death of *Cxxc1-null* mouse embryos prevented an assessment of Cfp1 function during later mammalian development and adult homeostasis. To address this question, mice were developed that are homozygous for a conditional *Cxxc1* allele and carry the *Mx1-Cre* recombinase transgene which can be induced in a wide range of tissues, particularly the liver and all hematopoietic cell lineages [21], [22], [23], [24]. Ablation of the *Cxxc1* gene in adult mice leads to a rapid loss of bone marrow progenitors and mature peripheral blood cells, and death within two weeks. Remarkably, the Lin^−^Sca-1^+^c-Kit^+^ (LSK) population of cells in the bone marrow, which is enriched for HSCs and multipotential progenitor cells (MPPs), persists and expands in the absence of Cfp1 during this time frame. In addition, Cfp1-deficient LSK cells exhibit neither reduced apoptosis nor increased proliferation. Overall, these findings indicate that Cfp1 is required for hematopoietic cell differentiation and/or the survival of differentiating cells.

## Materials and Methods

### Generation of a conditional *Cxxc1* allele

A conditional targeting vector based on the Cre-loxP system was constructed using the murine *Cxxc1* gene [25]. Linearized targeting vector was transfected into ES cells, and positive (G418)-negative (gancylovir) selection was performed. DNA from recovered ES clones was analyzed by Southern blot and PCR to identify homologous recombination events. PCR primers utilized in these studies include: LOXP1F, 5′-tgtagacacttgtgggaagcc-3′; LOXP1R, 5′-tgggctctatggcttctgagg-3′; LOXP3F, 5′-actgtttagccatctccc-3′; LOXP3R, 5′-gttcctgctcaaagagctcg-3′; CXXC1F, 5′-ttcgctgatcgttgcttccc-3′; and CXXC1R, 5′-agttcacccagaccctcttc-3′. An ES clone carrying the targeted *Cxxc1* allele was used for blastocyst injections and generation of chimeric animals. Mice heterozygous for the targeted *Cxxc1* allele were bred with mice carrying the *EIIa-Cre* transgene to remove the *Neo* cassette from intron 1 [26]. A mouse heterozygous for the conditional *Cxxc1* allele was backcrossed with C57BL/6J mice, and offspring carrying the conditional *Cxxc1* allele were bred with transgenic animals carrying the *Mx1-Cre* recombinase transgene [22].

### Ethics statement

This study was carried out in strict accordance with the recommendations in the Guide for the Care and Use of Laboratory Animals of the National Institutes of Health. The protocol was approved by the Institutional Animal Care and Use Committee of the Indiana University School of Medicine (Study Number: 3256). All efforts were made to minimize suffering.

### *In vivo* induction of the Cre recombinase and *ex vivo* culturing of primary bone marrow cells

Sex- and age-matched 6–12 week old mice were used for all experiments. To induce expression of the Cre recombinase, polyinosine-polycytosine (poly(I:C)) (GE Healthcare, Piscataway, NJ) was administered as three intraperitoneal injections (250 µg) at 2 day intervals [22], [27]. For ex vivo culturing, 1×10^6^ cells/ml of low density bone marrow cells (LDBMCs) were plated on 3.5 cm non-tissue culture dishes in IMDM medium supplemented with 20% FBS, 100 ng/ml thrombopoietin (TPO), 100 ng/ml stem cell factor (SCF), 50 ng/ml Flt-3 ligand, and 200 U/ml IL-6 (Peprotech, Rocky Hill, NJ) [27], [28].

### Immunoblots

Whole-cell lysates were prepared using 10 mM PIPES pH 7.0 containing 300 mM NaCl, 300 mM sucrose, 3 mM MgCl_2_, 1 mM EGTA, 0.5% Triton X-100, and protease inhibitors. Protein concentrations were determined by the Bradford protein assay, and immunoblots were performed as previously described [29]. Polyclonal anti-Cfp1 rabbit antiserum was used at a 1∶2500 dilution as previously described [14], and anti-actin monoclonal antibody was obtained from Sigma-Aldrich (St. Louis, MO).

### Histology

Tissues were collected in 10% neutral-buffered formalin. Bones were decalcified for 3 days prior to embedding (Formic Decalcifying Solution; US Biotex, Webbville, KY). Tissues were dehydrated, embedded, sectioned at 6 microns, and stained with hematoxylin and eosin.

### Hematopoietic cell analyses

Peripheral blood cell counts were determined from 50 microliter blood samples using a Hemavet analyzer. Spleen and bone marrow cell suspensions were prepared by flushing spleens or femurs with RPMI 1640 medium plus 10% FBS and 100 U/ml penicillin/streptomycin, and LDBMCs were prepared and progenitor colony forming activity was assayed as previously described [27], [28]. Briefly, LDBMCs were prepared by centrifugation on ficoll-hypaque (density 1.119; Histopaque-1119; Sigma –Aldrich). Cells were suspended in triplicate into 10×35 mm grid culture dishes with 1.2% methylcellulose (MethoCult M3134, StemCell Technologies; Vancouver, BC, Canada), 30% FBS, 1% deionized fraction V bovine serum albumin (Sigma-Aldrich), 0.1 mM ββ-mercaptoethanol, and cytokines (100 ng/ml recombinant murine IL-3 (Peprotech), 100 ng/ml recombinant human erythropoietin (Amgen; Thousand Oaks, CA), and 100 ng/ml recombinant murine SCF (Peprotech)), followed by 7 days of incubation at 37°C, 5% CO_2_ in a humidified incubator. Colony types were determined by observation using an inverted microscope and according to documented criteria [30].

For analysis of phenotypically defined hematopoietic cell populations, LDBMCs were isolated from 6–12 week old mice and suspended in IMDM medium supplemented with 10% FCS. To characterize LSK cells and subpopulations, 1×10^6^ cells were stained with an antibody cocktail composed of 0.125 microgram each of biotinylated antibodies for lineage markers CD11b/Mac-1 (M1/70), CD4 (RM4-5), CD8 (53-6.7), CD45R/B220 (RA3-6B2), Gr-1 (RB6-8C5), and TER119 (eBioscience Inc.; San Diego, CA) in 100 microliters of phosphate buffered saline (PBS) with 0.1% bovine serum albumin (BSA) on ice for 20 min. Samples were washed with PBS and 0.1% BSA, and stained with 0.5 microgram streptiavidin-Cy5.5 conjugated secondary antibody, 0.5 microgram PE-Cy7 conjugated anti-Sca-1, 0.5 microgram of allophycocyanin (APC)-conjugated anti-c-Kit, anti-Flt3/Flk2-PE, and anti-CD34-FITC antibodies (BD Pharmingen; San Diego, CA or eBioscience) on ice for 20 min, washed, and analyzed with a Becton Dickinson LSRII flow cytometer. To detect myeloid and lymphoid progenitors, LDBMCs were stained with 0.125 microgram each of biotinylated antibodies for the same lineage markers, and then stained with 0.5 microgram streptavidin-PE-Cy5.5 secondary antibody (eBioscience; San Diego, CA). Cells were stained with combinations of 0.5 microgram each of the indicated antibodies: anti-CD127/IL7Ra-APC-Alexa Fluor 750, anti-FcgRII/III-PE, anti-CD34-FITC, anti-Sca-1-PE-Cy7, and anti-c-Kit-APC monoclonal antibodies (BD Pharmingen or eBioscience) prior to flow cytometer analysis.

### Bone marrow transplantation studies

Bone marrow cells were isolated from *Cxxc1^flox/flox^Mx1-Cre* mutant mice or *Cxxc1^flox/flox^* mice lacking the *Mx1-Cre* transgene control donors, and 3×10^6^ LDBMCs were transplanted by tail vein injection into 6–12 week old wild type lethally irradiated recipients (1,100 cGy split dose) [31].

### Flow cytometric analysis of apoptosis and proliferation

Apoptosis assays were performed as previously described [28]. Briefly, freshly isolated LDBMCs or 1×10^5^ cells cultured ex vivo were harvested, washed once with PBS, and resuspended in 100 microliters of binding buffer (10 mM HEPES/NaOH pH 7.4, 140 mM NaCl, and 2.5 mM CaCl_2_). Cells were then stained with 5 microliters of AnnexinV-FITC (BD Pharmingen) and 500 ng of propidium iodide (PI) for in vitro cultured cells. When freshly isolated LDBMCs were analyzed, these cells were incubated with 5 microliters AnnexinV-FITC and 5 microliters (0.25 microgram) 7-AAD. Stained cells were incubated at room temperature for 15 min in the dark, and binding buffer (400 microliters) was then added prior to flow cytometric analysis using a FACScan flow cytometer. Apoptotic cells were defined as the fraction of Annexin V positive, and PI or 7-AAD negative cells.

To measure proliferation in vivo, poly(I:C) was administered as described above. Seven days after the first injection, 200 microliters of 10 mg/ml bromodeoxyuridine (BrdU) was administered by intraperitoneal injection. Twenty four hours later, LDBMCs were isolated and 1×10^6^ cells were stained with the antibodies listed above for lineage markers, and by 0.5 microgram streptavidin-APC-Cy7 secondary antibody, anti-Sca-1-PE-Cy7, anti-c-Kit-APC, anti-CD34-Pacific Blue, and anti-Flt3/Flk2-PE (BD Pharmingen and eBiosciences). Cells were then fixed, permabilized, and BrdU was detected in LSK cells and subpopulations using a FITC BrdU Flow Kit (BD Pharmingen) and a Becton Dickinson LSRII flow cytometer.

### Analysis of global genomic cytosine methylation levels

Genomic DNA was isolated from the indicated cell populations and the relative level of genomic cytosine methylation was assessed using a methyl-acceptance assay, as previously described [9].

## Results

### Generation of mice carrying a conditional *Cxxc1* allele

**Figure 1:**
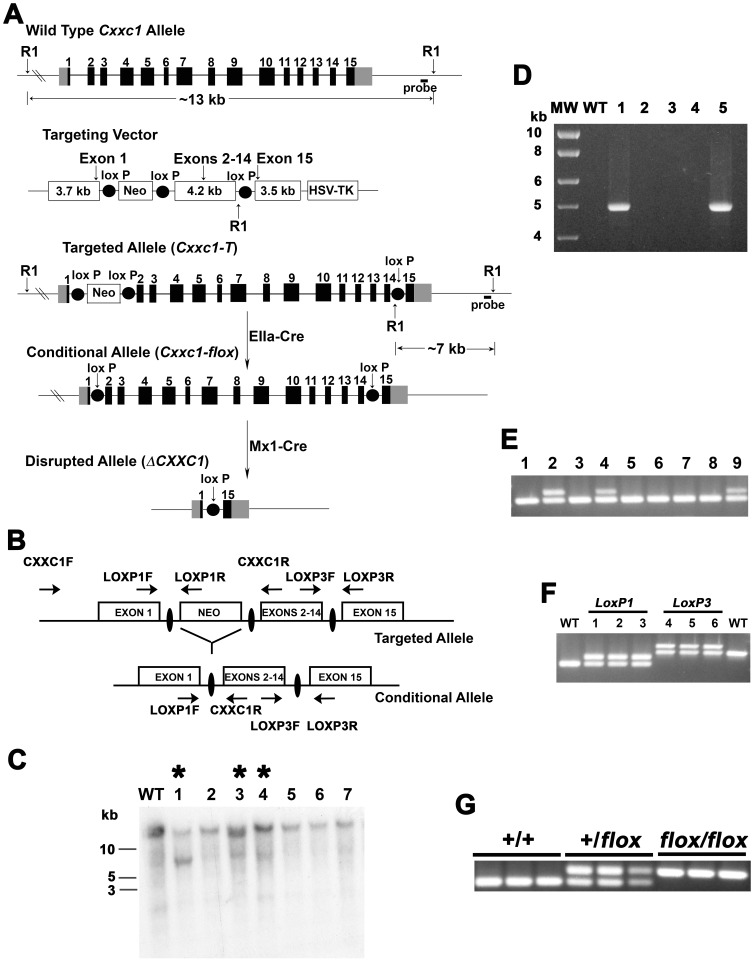
Generation of mice carrying conditional *Cxxc1* alleles. (A) Schematic illustrating structures of the endogenous murine *Cxxc1* allele, targeting vector, targeted *Cxxc1* allele (*Cxxc1-T*), conditional *Cxxc1* allele (*Cxxc1-flox*), and disrupted *Cxxc1* allele (*ΔCxxc1*). (B) Schematic illustrating the PCR primers used to detect the presence of loxP elements within *Cxxc1* alleles. (C) Southern blot analysis performed with *Eco*RI digested genomic DNA and a probe for a region of the *Cxxc1* locus downstream of the targeting construct detects a 7 kb fragment from the targeted allele (*Cxxc1-T*) and a 13 kb fragment from the wild type allele. The presence of the targeted allele is indicated by asterisks. (D) PCR using primers CXXC1F and LOXP1R demonstrates the presence of a loxP element within intron 1 of the *Cxxc1* allele in a subset of ES clones (lanes 1 and 5). (E) Mice carrying the targeted *Cxxc1* allele were bred with mice carrying the *EIIa-Cre* transgene. Tail DNA isolated from offspring was analyzed by PCR, using primers LOXP1F and CXXC1R, to detect Cre-mediated recombination events that removed the Neo cassette from intron 1 of the targeted *Cxxc1* allele, to produce the conditional *Cxxc1-flox* allele (indicated by the presence of the upper band of the doublet) (lanes 2, 4, and 9). (F) Following germline transmission of the putative conditional *Cxxc1-flox* allele, tail DNA of offspring was analyzed by PCR to confirm the presence of loxP elements within introns 1 and 14. Primers LOXP1F and CXXC1R were used to detect the intron 1 loxP element, and primers LOXP3F and LOXP3R were used to detect the loxP element within intron 14. In both cases the presence of the loxP element leads to the production of a band slightly larger than that produced from the wild type *Cxxc1* allele. In addition, the PCR fragment containing *LoxP1* was purified for DNA sequence analysis to confirm its presence and the absence of the *Neo^R^* cassette (data not shown). (G) PCR analysis, using primers LOXP1F and CXXC1R, was performed to identify mice lacking (+/+), heterozygous (+/flox), or homozygous (flox/flox) for the conditional *Cxxc1* allele.

Cfp1 is widely expressed [25], but the peri-implantation death of Cfp1-null mouse embryos prevented analysis of Cfp1 function later in mammalian development [12]. To determine the requirement of Cfp1 for homeostasis in an adult mammal, a mouse strain carrying a *Cxxc1* conditional deletion allele was generated (Figure 1A–E). An ES clone carrying the targeted *Cxxc1* allele was used for blastocyst injections and generation of chimeric animals, which were backcrossed with C57Bl/6J mice. Mice heterozygous for the targeted *Cxxc1* allele were bred with mice carrying the *EIIa-Cre* transgene in order to remove the *Neo^R^* cassette from intron 1 (Figure 1F) [26]. Mice carrying the conditional *Cxxc1* allele were bred with transgenic animals carrying the *Mx1-Cre* transgene (Figure 1G) to permit the poly(I:C)-inducible expression of Cre in a wide variety of tissues, including bone marrow cells [22], [27], [32], [33].

### Deletion of the *Cxxc1* gene causes hematopoietic failure and death

**Figure 2:**
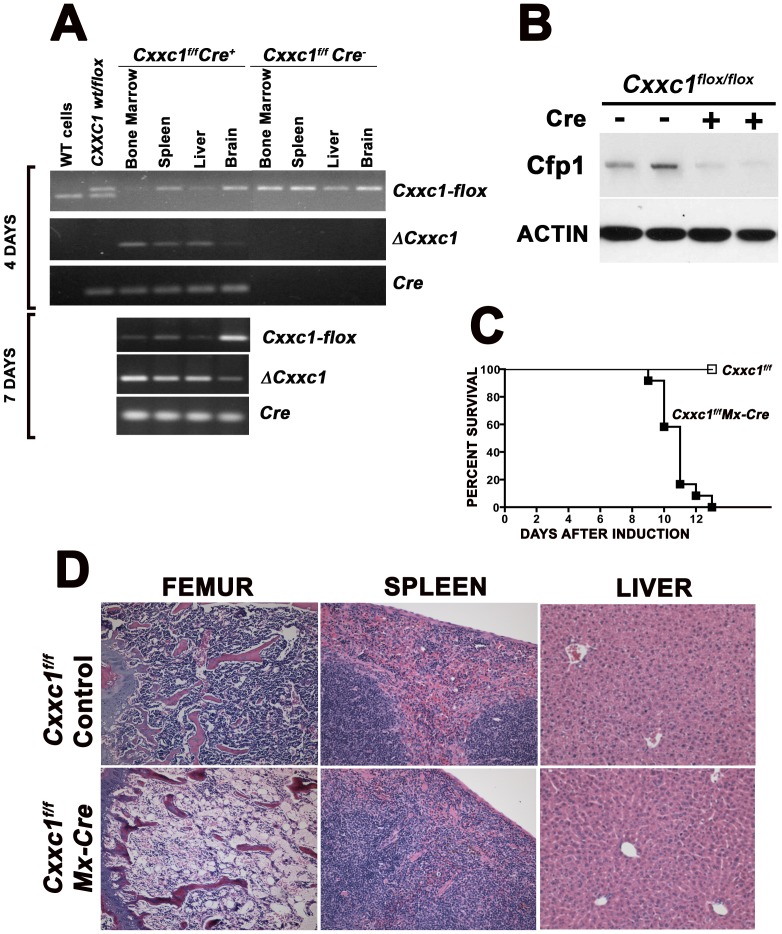
Ablation of the *Cxxc1* gene in adult mice causes death. Mice homozygous for the conditional *Cxxc1* allele and carrying or lacking the *Mx1-Cre* transgene were injected with poly(I:C) at days 0, 2, and 4. (A) Indicated tissues were collected at day 4 or day 7 following the initiation of Cre induction. Genomic DNA was isolated and PCR analysis was performed to assess the relative abundance of the conditional *Cxxc1* allele (*Cxxc1-flox*) and recombined *Cxxc1* (*ΔCxxc1*) allele, as well as the presence of the *Mx1-Cre* transgene (*Cre*). (B) Bone marrow cells were collected 48 hours after a single poly(I:C) injection and cultured ex vivo for 2 days. Cellular extracts were analyzed for Cfp1 protein levels by western analysis. Actin levels were determined as a loading control. (C) Animals were observed for survival following initiation of Cre induction. (N = 12 for each genotype) (D) Light microscopy of femur, spleen, and liver collected 11 days after initiation of Cre induction. There is a marked depletion of hematopoietic cells in the bone marrow and reduction of red pulp in the spleen of mutant mice. The liver appears normal.

To determine the effect of Cfp1-deficiency in an adult animal, *Cxxc1^flox/flox^ Mx1-Cre+* (mutant) and *Cxxc1^flox/flox^* mice lacking the *Mx1-Cre* transgene (control) were induced with poly(I:C) to stimulate expression of the Cre recombinase. Previous reports demonstrate that induction of the *Mx1-Cre* transgene causes efficient deletion of floxed genes in the liver, spleen, and duodenum (70–100%), deletion to a lesser extent in heart, lung, uterus, thymus, and kidney (40–50%), even less in muscle and tail (20%), and very little in the brain (less than 10%) [22]. Also, numerous groups, including ours, demonstrated effective deletion of floxed genes in the bone marrow following induction of the *Mx1-Cre* transgene [27], [32], [33]. As shown in figure 2A, within 4 days after the initiation of Cre induction, significant deletion of the conditional *Cxxc1* gene (Δ*Cxxc1*) was detected in bone marrow cells and liver of *Cxxc1^flox/flox^* mice carrying the *Mx1-Cre* transgene, but not in control animals, and Cfp1 protein was nearly undetectable in bone marrow cells isolated from mutant mice (Figure 2B). In agreement with previous data [22], little Cre-mediated *Cxxc1* deletion was detected in the brain. While control animals remained healthy, mutant mice died between 9 and 13 days after the initiation of poly(I:C) injections (Figure 2C), with more than 80% dying between 9 and 11 days. Mice carrying the *Mx1-Cre* transgene but otherwise wild type, as well as heterozygous *Cxxc1^flox/wt^ Mx1-Cre* mice, all remained healthy following induction with poly(I:C).

Necropsies were performed to identify which organ(s) fail coincident with loss of survival. At 5 and 8 days following Cre induction a loss of cellularity was apparent in the bone marrow and in the red pulp of the spleen of mutants, with occasional apoptotic cells observed. The white pulp of the spleen appeared the same in mutants and controls. In the small intestine, occasional apoptotic cells were observed in the crypts of mutants, but villus height was unaffected. Loss of bone marrow cellularity was more severe at 8 days. In the spleen, extensive accumulation of hemosiderin, likely indicating uptake and degradation of red blood cell precursors, was observed. By 11 days the loss of cellularity in the bone marrow and red pulp of the spleen in mutants was much more severe (Figure 2D). No pathology was observed in other tissues, including liver, which exhibits an efficiency of Cre-mediated *Cxxc1* gene ablation similar to that found in bone marrow (Figure 2A, D).

**Figure 3:**
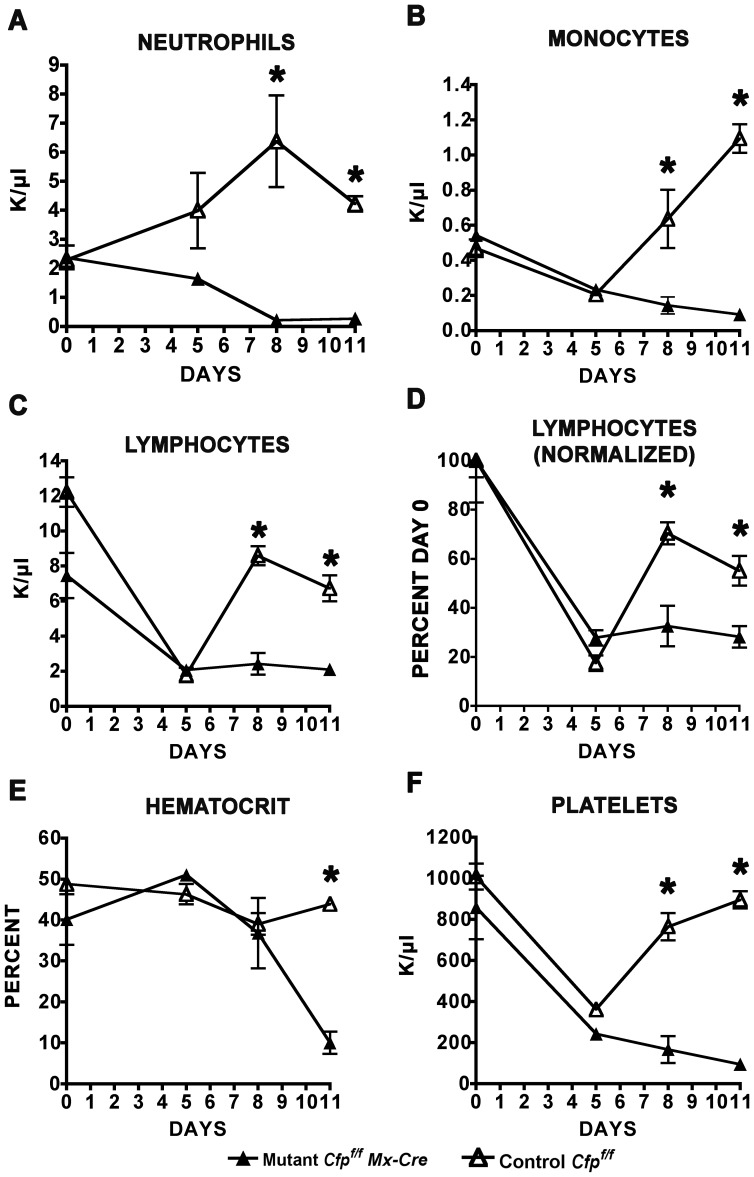
Peripheral blood cells are depleted following ablation of the *Cxxc1* gene. Mice homozygous for the conditional *Cxxc1* allele and carrying or lacking the *Mx1-Cre* transgene were injected with poly(I:C) at days 0, 2, and 4. Peripheral blood cell counts were determined at days 0, 5, 8 and 11 following the initiation of Cre induction for (A) neutrophils, (B) monocytes, (C and D) lymphocytes, (E) red blood cells (hematocrit), and (F) platelets. Open triangles, *Cxxc1^f/f^ Cre* ^−^ control mice; filled triangles, *Cxxc1^f/f^ Cre^+^* mice. Asterisks denote a statistically significant reduction in cell numbers following ablation of the *Cxxc1* gene (P<0.02). N = 3–7 for each of two independent experiments.

Given the striking loss of cellularity in hematopoietic organs following ablation of the *Cxxc1* gene, we examined the frequency of mature hematopoietic cells in the peripheral blood, which were found to decline sharply following *Cxxc1* gene deletion (Figure 3). Neutrophil counts were reduced as early as 5 days after the initiation of Cre induction and reached a minimum by 8 days. Cell counts for monocytes and lymphocytes declined initially following poly(I:C) injection in both mutant and control animals due to the interferon response, as previously observed [34], [35], but while control counts rebounded, mutant counts remained low. The lymphoid counts were normalized to day 0 due to differences in the steady state levels of lymphoid counts in uninduced mutant and control animals. This difference is likely due to a low level of leakiness from the *Mx1-Cre* transgene, as was previously reported [36]. Hematocrits in mutants declined from 40–50% to less than 10% by day 11. Platelet counts for mutants and controls declined initially at day 5, but mutant counts continued to decline to only 100 K/µl by day 11, while control counts recovered. In summary, these findings indicate that deletion of the *Cxxc1* gene results in hematopoietic failure and death.

### Deletion of the *Cxxc1* gene in the bone marrow is sufficient to cause apoptosis, hematopoietic failure, and death

**Figure 4:**
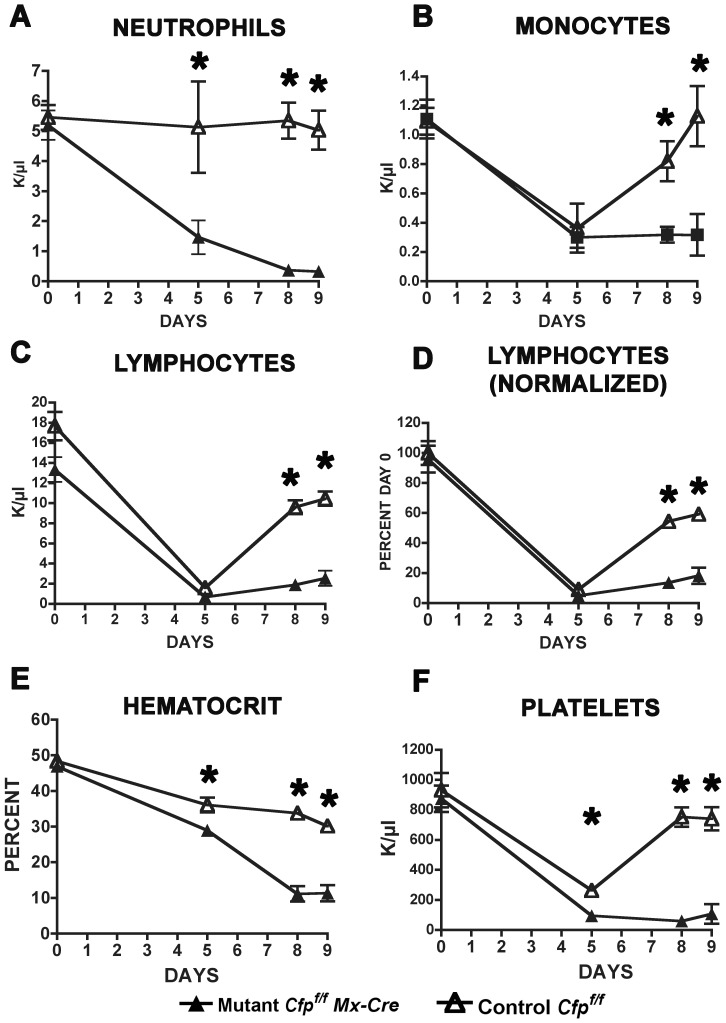
Hematopoiesis failure following *Cxxc1* gene ablation is intrinsic to bone marrow cells. Bone marrow was harvested from mice homozygous for the conditional *Cxxc1* allele and either carrying or lacking the *Mx1-Cre* transgene and used to transplant lethally-irradiated wild type recipient mice. Following a several month period for engraftment, transplanted mice were injected with poly(I:C) at days 0, 2, and 4, and peripheral blood cell counts were determined at the indicated days following initiation of Cre induction as described for Figure 3. Open triangles, *Cxxc1^f/f^ Cre* ^−^ control mice; filled triangles, *Cxxc1^f/f^ Cre^+^* mice. Asterisks denote a statistically significant reduction in cell numbers following ablation of the *Cxxc1* gene (p≤0.02). N = 5 for recipients of mutant bone marrow and N = 6 for recipients of control bone marrow for each of two independent experiments.

Additional studies were performed to assess whether the observed failure of hematopoiesis and subsequent death following *Cxxc1* deletion reflects a Cfp1 function intrinsic to cells in the bone marrow. Bone marrow cells were isolated from mutant or control mice and transplanted separately into lethally-irradiated wild type recipients. The donor cells were allowed to engraft for 2–3 months, and then Cre-mediated deletion of the *Cxxc1* gene was induced with poly(I:C). Unexpectedly, the neutrophil, monocyte, and lymphocyte counts for recipients of both mutant and control cells were initially elevated compared to non-transplanted mutant and control mice (Figures 3 and 4). Importantly, however, blood counts for all recipients of mutant bone marrow declined to similar levels and with similar kinetics to those of induced *Cxxc1^flox/flox^ Mx1-Cre* mutant mice (Figure 4). Further, these recipients all died 9 days after induction, also similar to the mutant mice (Figure 2C). The recipients of control bone marrow remained healthy, with blood counts comparable to those of *Cxxc1^flox/flox^* mice lacking the *Mx1-Cre* transgene.

**Figure 5:**
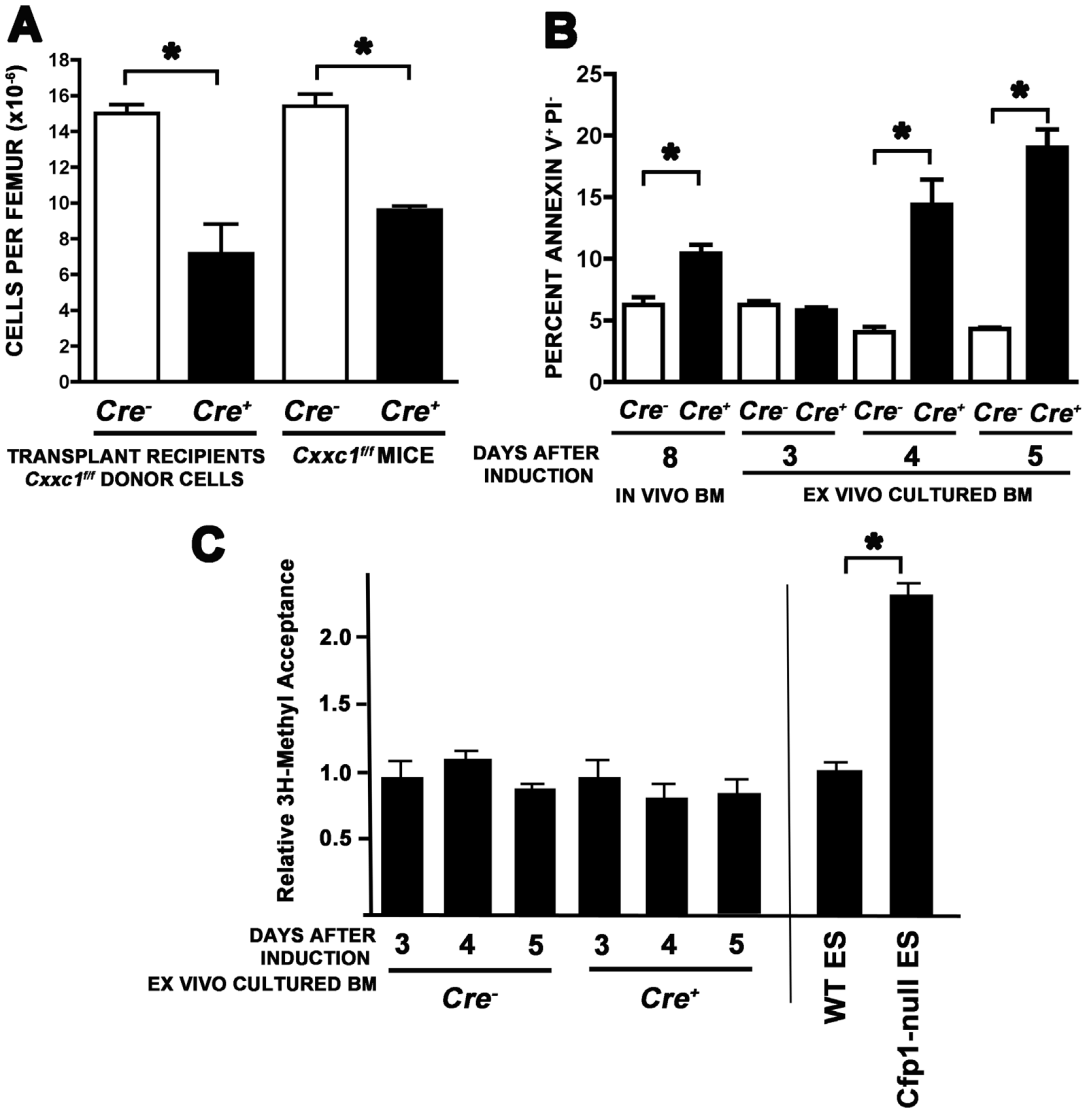
Bone marrow exhibits decreased cellularity and increased apoptosis, but no change in global cytosine methylation, following ablation of the *Cxxc1* gene. (A) Following transplantation of control or mutant bone marrow (N = 2 and N = 3 recipients, respectively), poly(I:C) was injected on days 0, 2, and 4, and on day 9 following initiation of Cre induction total bone marrow cells were isolated and analyzed (P = 0.04). Similarly, control or mutant mice were induced with poly(I:C), and on day 8 following initiation of Cre induction, total bone marrow was analyzed; N = 11 controls and N = 16 mutant mice (P<0.0001). (B) Bone marrow cells were collected from mice of the indicated genotypes on day 8 after day 0, 2, and 4 poly(I:C) injections, and cells were analyzed for apoptosis by flow cytometry using Annexin V and PI staining (“in vivo BM”); N = 6 for Cre- and N = 5 for Cre+ for each of two independent experiments. Alternatively, after a poly(I:C) injection on day 0, bone marrow was collected on day 2 from mice of the indicated genotypes, cultured ex vivo, and then analyzed for apoptosis by Annexin V and PI staining at days 3, 4, and 5 following initiation of Cre induction (“in vitro cultured BM”); for each genotype, N = 4 for day 3 and N = 7 for days 4 and 5 (P≤0.0003) for each of two independent experiments. Asterisks denote statistically significant differences in cell number or frequency in total LDBMCs. (C) Genomic DNA was isolated from ex vivo cultured bone marrow cells as described in (B). Global genomic cytosine methylation levels were determined using a methyl-acceptance assay, as previously described [9]. Similar analysis was done using DNA isolated from wild type or Cfp1-null ES cells as controls. Asterisk denotes a statistically significant difference (P<0.001).

Upon *Cxxc1* gene deletion, the loss of bone marrow cellularity in mice transplanted with mutant bone marrow resembled that observed in *Cxxc1^flox/flox^Mx1-Cre* mice (Figure 5A). The bone marrow cellularity of mice transplanted with mutant cells was 48% of controls (7.2×10^6^ cells per femur compared with 15×10^6^) at 9 days after induction of *Cxxc1* gene deletion. Similarly, at 8 days after initiation of deletion, bone marrow cellularity in mutant *Cxxc1^flox/flox^Mx1-Cre* mice was 9.6×10^6^ cells per femur, a decline to 62% of controls (15.4×10^6^ cells per femur). Overall, these findings indicate that the death of *Cxxc1^flox/flox^ Mx1-Cre* mice after *Cxxc1* gene deletion resulted from a bone marrow cell-intrinsic Cfp1 deficiency and the ensuing hematopoietic failure.

The striking loss of cells in the bone marrow and red pulp of the spleen, and the coincident observation of infrequent apoptotic cells in these tissues, suggests that Cfp1-deficiency might cause apoptosis. Therefore, *Cxxc1* deletion was induced in mutant mice, and the presence in the bone marrow of Annexin-V positive, 7-AAD negative staining cells undergoing apoptosis was detected 8 days after Cre induction and compared with that for identically treated controls. As shown in Figure 5B, the frequency of apoptotic cells increased 66% (P = 0.0015) in the bone marrow of mutants compared to controls, indicating that increased programmed cell death likely contributes to the loss of bone marrow cells after *Cxxc1* deletion.

Since apoptotic cells in the bone marrow are rapidly subjected to phagocytosis by macrophages [37], the data derived from analysis of bone marrow cells is likely an underestimate of the level of apoptosis that occurs following *Cxxc1* gene deletion. As an alternative approach, *Cxxc1* deletion was induced in vivo, and then the frequency of bone marrow cells undergoing apoptosis was detected at various times following ex vivo culturing (Figure 5B). Approximately 4-fold more apoptotic cells were detected in cultures of induced mutant cells than for control cells 4 and 5 days after induction (P = 0.0003 and P<0.0001, respectively). Surprisingly, and in contrast to the situation in *Cxxc1-null* ES cells [9], methyl-acceptance assays failed to detect reduced levels of global genomic 5-methylcytosine in Cfp1-deficient total bone marrow cells that lack Cfp1 and exhibit elevated rates of apoptosis (Figure 5C). Overall, these findings further indicate the requirement of Cfp1 for bone marrow cell survival.

### *Cxxc1* gene deletion causes loss of lineage-restricted hematopoietic progenitors but not populations enriched for hematopoietic stem and primitive progenitor cells

**Figure 6:**
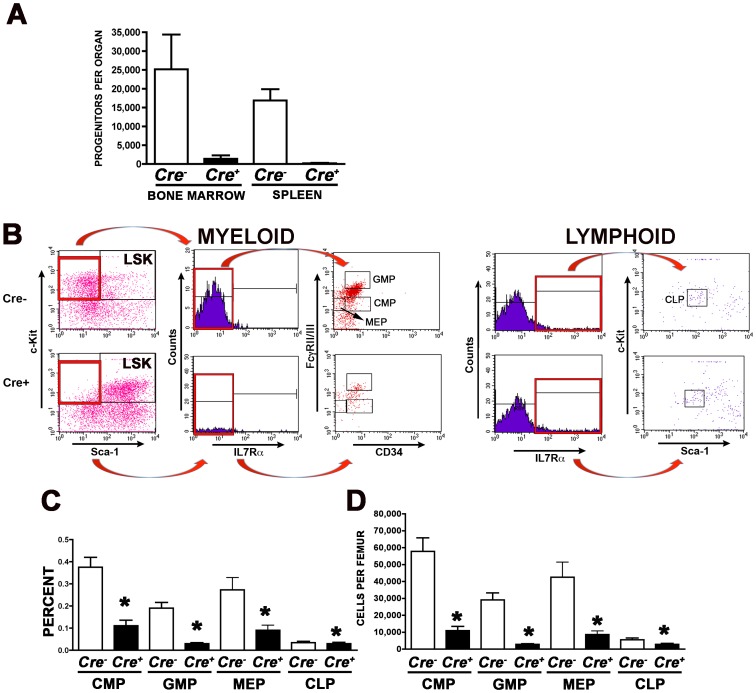
Hematopoietic progenitor cell populations are sensitive to Cfp1 depletion. Mutant and control mice were induced with poly(I:C) injections at days 0, 2, and 4. Tissues were collected on day 8 following initiation of Cre induction for all analyses. (A) Bone marrow and spleen cells were each collected and analyzed by colony forming assay for myeloid hematopoietic progenitors. For both tissues, N = 3 for Cre- controls and N = 8 for Cre+ mutants. (B–D) Bone marrow cells were collected, stained for the indicated cell surface markers, and analyzed by flow cytometry. (B) Scatter plot showing a representative experiment. (C) Summary for frequency per total LDBMCs (percent) of CMP (Lin- Sca1- Kit+ IL7Ra- FcgRII/III lo CD34+), GMP (Lin- Sca1- Kit+ IL7Ra- FcgRII/III hi CD34+), MEP (Lin- Sca1- Kit+ IL7Ra- FcgRII/III lo CD34-), and CLP (Lin- Sca1 lo Kit lo IL7Ra+). (D) Summary for number of stained cells per femur. (For C–D, N = 11 for Cre- controls and N = 16 for Cre+ mutants for three independent experiments.) Asterisks denote statistically significant differences in cell number or frequency (P≤0.03).

Additional studies were conducted to investigate at what stage(s) of ontogeny hematopoietic cells are lost following *Cxxc1* gene deletion. Assays were performed to quantify hematopoietic progenitors following *Cxxc1* gene deletion. Progenitor methylcellulose colony assays revealed that few functional myeloid progenitors remained by 8 days post-Cre induction (Figure 6A). Bone marrow progenitors exhibited an 18-fold decline following Cre induction, and spleen progenitors declined 85-fold (Figure 6A, P = 0.016 and P<0.0001, respectively). Consistent with the loss of all lineages of mature cells in the peripheral blood (Figure 3), progenitors for all lineages assayed (erythroid, granulocyte, macrophage) were dramatically reduced, as well as more primitive colony-forming unit-mixed lineage (granulocyte-erythrocyte-macrophage-megakaryocyte) progenitors (CFU-GEMM).

For this functional assay, progenitors must differentiate and form a colony of mature hematopoietic cells to be counted. Progenitor cells that might have been present but unable to proliferate and differentiate would not have been detected. Our previous studies established that Cfp1-deficient ES cells are unable to differentiate in vitro [9], so if Cfp1-deficiency also impairs the ability of hematopoietic progenitors to differentiate, the methylcellulose colony assay would fail to detect these progenitors. Therefore, immunophenotypic analysis was used to identify populations of cells enriched for various types of hematopoietic progenitors. *Cxxc1* gene deletion was induced in *Cxxc1^flox/flox^Mx1-Cre* mice and controls, and 8 days later LDBMCs were isolated and the frequency and abundance of common myeloid progenitors (CMP), granulocyte-macrophage progenitors (GMP), megakaryocyte-erythroid progenitors (MEP), and common lymphoid progenitors (CLP) were determined by detecting the relevant immunophenotypic markers (Figure 6B–D). The frequency per total LDBMCs of each of these progenitor populations was significantly reduced following *Cxxc1* gene ablation. CMP frequency declined 3.4-fold, GMP declined 6.4-fold, and MEP fell 2.8-fold (P<0.0001). CLP frequency declined slightly but not by a significant amount. However, because of reduced overall bone marrow cellularity, these reductions appear greater when expressed as number of progenitor cells per femur (Figure 6D), and a significant 2.0-fold reduction in CLP number became apparent (P = 0.03). Myeloid progenitor numbers declined 4.9- to 10.2-fold (P<0.0001). These findings indicate that the reduction in myeloid progenitors detected in colony forming assays results from a loss of progenitors and possibly their inability to differentiate as well. Taken together, these data indicate that Cfp1-deficiency results in the loss of both myeloid and lymphoid progenitors.

**Figure 7:**
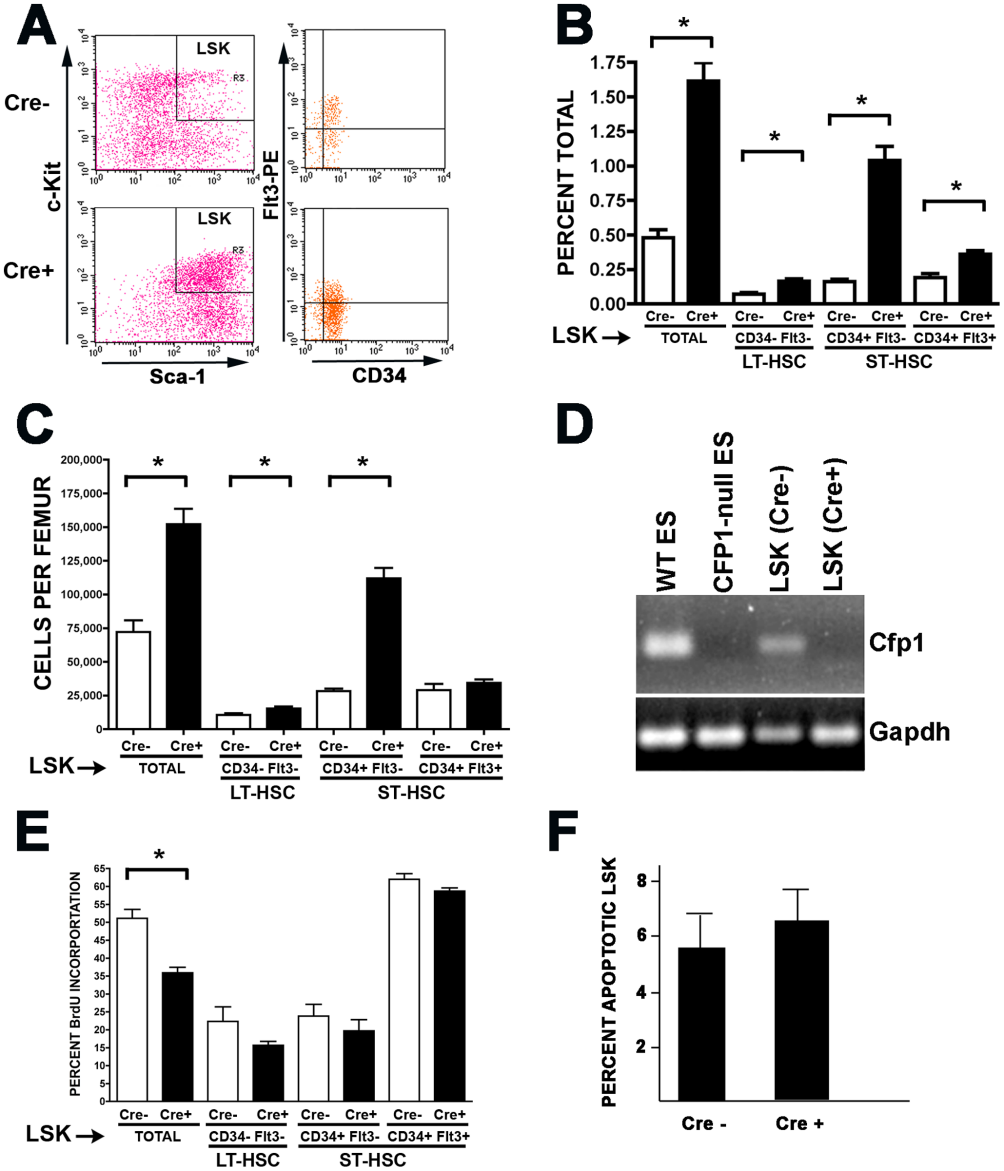
Hematopoietic cells enriched for stem cells are tolerant of Cfp1 depletion. Mutant and control mice were induced with poly(I:C) injections at days 0, 2, and 4. Bone marrow cells were collected at day 8 following initiation of Cre induction and analyzed by flow cytometry for the indicated cell surface markers. (A) Scatter plot showing a representative experiment. (B–C) Summary for enumeration of LSK (Lin- Sca1+ Kit+), LT-HSC (Lin- Sca1+ Kit+ CD34- Flt3-), ST-HSC (Lin- Sca1+ Kit+ CD34+ Flt3+), and more primitive ST-HSC (Lin- Sca1+ Kit+ CD34+ Flt3-). (A) and (B) present the data in terms of percent of total LDBMCs, while (C) presents cell number per femur. (For B–C, N = 11 for Cre- controls and N = 16 for Cre+ mutants for each of three independent experiments.) (D) Total RNA was isolated from purified LSK cells derived from the bone marrow of mice of the indicated genotypes (day 8 after poly(I:C) injections on days 0, 2, and 4), and semi-quantitative RT-PCR was performed to determine the level of *Cxxc1* transcript. RNA from wild type or *Cxxc1-null* ES cells was used as positive and negative controls, respectively [9]. The conditions for all PCR reactions were confirmed to be within the linear range (data not shown). (E) Mutant and control mice were induced with poly(I:C) injections on days 0, 2, and 4, and BrdU was injected on day 7. On day 8 following initiation of Cre induction, bone marrow was isolated and analyzed for the frequency of BrdU incorporation into LSK cells and CD34/Flt3 subpopulations. Asterisks denote statistically significant differences (P≤0.02). (F) LSK cells were recovered from the bone marrow of mutant or control mice 8 days following Cre induction and examined for apoptosis by Annexin V and PI staining.

To investigate the requirement for Cfp1 in more primitive hematopoietic cells, the frequency of a phenotypically defined population of Lin- Sca+ c-Kit+ (LSK) cells, enriched for HSCs and MPPs [38], [39] was determined by flow cytometric analysis of mutant or control LDBMCs isolated 8 days after induction of Cre recombinase. The frequency and number per femur of LSK cells in mutant animals was 3.4-fold and 2-fold greater than controls, respectively (Figure 7A–C, P<0.0001), in striking contrast with the marked loss of progenitors and mature cells at this time point. Analysis of further-refined bone marrow cell populations revealed that cells enriched with long term HSCs (LSK, CD34- Flt3-) or short term HSCs (LSK, CD34+ Flt3+) both exhibit increases in cell frequency following *Cxxc1* gene ablation (2.3- and 1.9-fold increases, respectively, with P<0.0001, Figure 7B) [40]. However, when the number of cells per femur for each population was considered, increases following *Cxxc1* deletion were smaller. The 1.4-fold increase for LSK, CD34-Flt3- cell number was statistically significant (P = 0.02), but that for the LSK, CD34+Flt3+ population was not (Figure 7C). These findings are consistent with the loss of predominantly more mature bone marrow cells following *Cxxc1* ablation and the preservation of more primitive cells. Remarkably, the frequency and number of an intermediate cell population (LSK, CD34+ Flt3-), which is thought to represent a more primitive form of short term HSCs [41], was dramatically elevated following ablation of the *Cxxc1* gene (6.4-fold and 4.0-fold, respectively; P<0.0001), and the increase of this population accounts for the majority of the overall increase of mutant LSK cells (Figure 7B–C). Further, we detected *Cxxc1* expression in LSK cells, and this transcript is lost upon *Cxxc1* deletion (Figure 7D), Thus, the tolerance of these cells to Cfp1 loss is not due to *Cxxc1* normally being absent in these cells or to the failure of the Cre recombinase to induce *Cxxc1* deletion in these cell populations.

An increase in proliferation and/or a decrease in apoptosis of Cfp1-deficient LSK cells might contribute to their increase in number. To measure proliferation of these cells in vivo, *Cxxc1* deletion was induced in *Cxxc1^flox/flox^Mx1-Cre* mice, and then BrdU incorporation into DNA was quantified and compared to identically treated control animals. Poly(I:C) was administered to mutant and control animals on days 0, 2, and 4 and then 2 mg of BrdU was administered on day 7 by peritoneal injection. Twenty-four hours later on day 8, bone marrow was isolated and analyzed for the frequency of BrdU incorporation into LSK cells and subpopulations. BrdU incorporation in LSK cells from mutant animals was not increased compared to controls. Instead there was a modest, but significant decrease in proliferation, with 36% of the mutant LSK cells incorporating BrdU compared with 51% of the control cells (Figure 7E, p = 0.0004). Similar but smaller trends were observed for CD34/Flt3 subpopulations of LSK cells, but the differences between mutant and control animals were not significant. LSK cells from mutant and control mice were also examined for apoptosis 8 days after poly(I:C) injections were initiated. With 6.3% of LSK cells Annexin V^+^, 7-AAD^−^ for mutant mice compared with 5.6% for controls, there was no significant difference for LSK cells undergoing apoptosis (Figure 7F). Together, these results indicate that neither an increase in proliferation nor a decrease in apoptosis contribute to the increase in number and frequency of LSK cells that follows *Cxxc1* deletion. Thus, while mature hematopoietic cells and lineage-committed progenitors are lost in animals following *Cxxc1* gene ablation, the population of HSCs and primitive progenitor cells expands and is tolerant of Cfp1 depletion.

### The accumulation of cells enriched for hematopoietic stem and primitive progenitor cells following *Cxxc1* deletion is distinct from transient interferon effects

**Figure 8:**
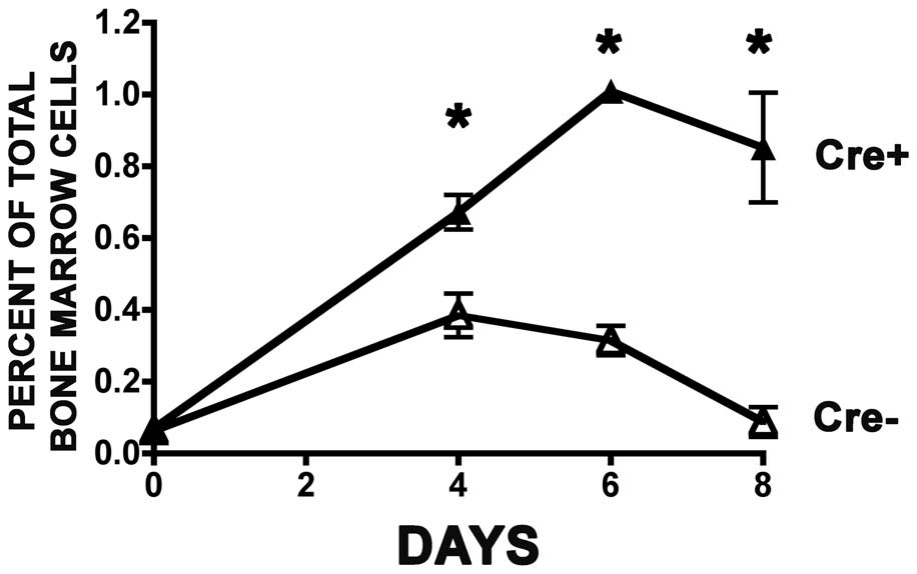
The accumulation of LSK CD150+ cells following *Cxxc1* deletion is distinct from transient interferon effects. Mutant and control mice were induced with poly(I:C) injections on days 0, 2, and 4, and bone marrow was analyzed by flow cytometry for LSK-CD150+ frequency on days 4, 6, or 8 following initiation of Cre induction. N = 4 or 5 for each genotype and each time point. Asterisks denote statistically significant differences (P≤0.01).

Because poly(I:C) affects hematopoiesis [34], [35], it was important to compare any effects on hematopoiesis resulting from poly(I:C)-induced *Cxxc1* deletion to poly(I:C) treated controls. This was the case for all of the experiments described in this report. In addition, LSK frequency was assessed in a time course to gauge the extent of an interferon effect on LSK frequency at the time when the effects of *Cxxc1* deletion were measured (day 8). On days 0, 2, and 4, poly(I:C) was administered to mutant and control mice. On days 4, 6, and 8 bone marrow was isolated and LSK frequency was determined. The presence of CD150, which further enriches for HSCs, was also determined [42]. As shown in Figure 8, there was a transient increase in LSK CD150+ frequency in control animals that peaked at day 4 and returned to the initial level by day 8. In contrast, LSK CD150+ frequency in *Cxxc1* mutants increased more than 14-fold by day 6 and remained elevated at day 8. At days 4, 6, and 8, LSK CD150+ frequency following Cfp1 depletion was 1.7-, 3.2-, and 9.7-fold greater than that of the control. Therefore, there was a transient increase in LSK CD150+ cells caused by poly(I:C) itself, but a much greater and sustained increase in these cells resulted from Cxxc*1* deletion.

## Discussion

Cfp1 is a ubiquitously expressed regulator of both cytosine and histone methylation, and Cfp1-deficient mouse embryos fail to develop past gastrulation. Cfp1-null ES are viable but unable to differentiate in vitro, suggesting an essential role for Cfp1 in regulating chromatin states during development. Mice carrying a conditional deletion allele of the *Cxxc1* gene were developed to analyze Cfp1 function in adult animals. The studies reported here demonstrate that acute depletion of the epigenetic regulator Cfp1 in an adult animal leads to death within two weeks as a consequence of hematopoiesis failure, demonstrating that Cfp1 is required for the survival of differentiating hematopoietic cells. A recent survey of 425 chromatin-associated factors documented that multiple components of the zebrafish Set1 complex, including Cfp1, are required for definitive hematopoiesis [43]. Together with previous findings from our laboratory that Cfp1 is required for primitive hematopoiesis during zebrafish embryogenesis [19] and for the survival of a human leukemia cell line [20], these results demonstrate that Cfp1 is required for the survival of differentiating hematopoietic cells.

The ability to ablate the *Cxxc1* gene in an adult animal offers an opportunity for detailed study of the sensitivity of cells to Cfp1 depletion at various stages of ontogeny. Remarkably, despite a nearly complete loss of lineage-committed progenitors and mature hematopoietic cells, bone marrow LSK cells enriched for HSCs and MPPs persist and expand after *Cxxc1* deletion. Many other investigators have utilized the *Mx1-Cre* transgene system to mediate target gene ablation in HSCs and MPPs [44], [45], [46], [47], [48], [49], and our data confirm Cre-mediated depletion of the *Cxxc1* transcript in LSK bone marrow cells. Thus, these data reveal that while Cfp1 is required in hematopoietic progenitors undergoing differentiation and lineage-commitment, HSCs and MPPs are tolerant of its depletion. The finding of an expanded LSK compartment in the bone marrow following Cfp1 depletion, coincident with the nearly complete loss of progenitor cells and mature peripheral blood cells, is unusual and highlights the distinct epigenetic requirements for HSC/MPP maintenance and differentiation programs.

The observed expansion of LSK cells upon loss of Cfp1 is associated with decreased cell proliferation and no significant change in the rate of apoptosis, suggesting that these cells exhibit a defect in differentiation. The accumulation of a primitive form of short term HSCs (CD34+ Flt3-) in the bone marrow following Cfp1 depletion suggests a differentiation block at an early stage of hematopoietic development. This is consistent with previous work that showed differentiation defects in Cfp1-null ES cells as well as Cfp1-null blastocysts [9], [12]. We also demonstrated previously that ES cells lacking Cfp1 exhibit decreased levels of cytosine methylation and increased levels of histone H3-Lys4 methylation, indicating reduced levels of heterochromatin in these cells [9], [13], [14]. Because heterochromatin formation is necessary for lineage commitment and restriction of developmental potential of stem cells [5], [6], taken together, these findings suggest that Cfp1 is required to facilitate remodeling of chromatin structure that is associated with stem cell differentiation. We cannot exclude the possibility that expansion of LSK cells following loss of Cfp1 reflects inappropriate induction of stem cell markers in non-stem cells as a consequence of perturbed epigenetic regulation of gene expression.

Modulation of a number of chromatin regulators has been shown to expand HSC number and/or activity. For example, loss of appropriate chromatin structure is implicated in the phenomenon of HSC exhaustion following replicative stress. But this can be prevented by over-expression of the Polycomb group protein Ezh2, a regulator of histone methylation and deacetylation [50]. Furthermore, treatment of HSCs with inhibitors of DNA methyltransferases (azacytidine) and histone deacetylases (trichostatin A, valproic acid) leads to altered cell fate and an expansion ex vivo of HSCs that retain bone marrow-repopulating potential [51], [52], [53], [54], [55].

Cfp1 physically interacts with Dnmt1 [10] and facilitates maintenance cytosine methylation, and a decline in global genomic cytosine methylation is the most obvious molecular defect observed in ES cells depleted of Cfp1 [9], [19]. However, apoptosis of hematopoietic cells following Cfp1 depletion occurs without a change in global cytosine methylation levels. Furthermore, ablation of a conditional *Dnmt1* gene leads to hematopoietic failure associated with a rapid loss of LSK cells [56], in sharp contrast with the expansion of LSK cells that occurs following ablation of the *Cxxc1* gene. Thus, the hematopoietic failure observed upon Cfp1 depletion does not appear to be a consequence of altered Dnmt1 function.

Interestingly, similar to the Cfp-1 null phenotype, *Mx1-Cre* driven ablation of the murine de novo DNA methyltransferase *Dnmt3a* gene leads to an expansion of the HSC compartment and coincident decline in differentiation potential following serial bone marrow transplantations [57]. Also similar to ex vivo Cfp1-null bone marrow cells, Dnmt3a-null HSCs don’t exhibit altered global levels of genomic cytosine methylation, although gene-specific hyper- and hypomethylation were detected, including hypomethylation and incomplete repression of HSC-specific genes. It should be noted that global de novo DNA methyltransferase activity is normal in Cfp1-null ES cells, although the relative contributions of the Dnmt3a and Dnmt3b de novo methyltransferases were not distinguished [9]. The similarity of the knock-out phenotypes may suggest a previously unrecognized functional interaction between Cfp1 and Dnmt3a. Thus, further gene-specific interrogation of cytosine methylation patterns in Cfp1-null HSCs might be informative.
